# Classical QSAR and Docking Simulation of 4-Pyridone Derivatives for Their Antimalarial Activity

**DOI:** 10.3390/molecules23123166

**Published:** 2018-12-01

**Authors:** Máryury Flores-Sumoza, Jackson J. Alcázar, Edgar Márquez, José R. Mora, Jesús Lezama, Esneyder Puello

**Affiliations:** 1 Laboratorio de Fisicoquímica Orgánica y Química Computacional, Escuela de Ciencias, Departamento de Química, Universidad de Oriente, Cumaná 6001, Venezuela; maryuryfs@yahoo.com (M.F.-S.); jlezgar@gmail.com (J.L.); 2 Departamento de Química, Pontificia Universidad Católica de Chile, Casilla 306, Santiago 6094411, Chile; jacksonalcazar@gmail.com; 3 Grupo de Investigaciones en Química y Biología, Departamento de Química y Biología, Facultad de ciencias Básicas, Universidad del Norte, Carrera 51B, Km 5, vía Puerto Colombia, Barranquilla 081007, Colombia; 4 Grupo de Química computacional y teórica (QCT-USFQ) & instituto de Simulación Computacional (ISC-USF) Colegio Politécnico de Ciencias e Ingeniería Diego de Robles, y Vía Interoceánica, Universidad San Francisco de Quito, Quito 170901, Ecuador; jrmora@usfq.edu.ec; 5 Grupo de Investigación en Oxi/Hidrotratamiento Catalítico y Nuevos Materiales, Programa de Química-Ciencias Básicas, Universidad del Atlántico, Barranquilla 081001, Colombia; snypollqco@yahoo.com

**Keywords:** computational study, DFT, nitrogen compounds, molecular descriptors

## Abstract

In this work, the minimum energy structures of 22 4-pyridone derivatives have been optimized at Density Functional Theory level, and several quantum molecular, including electronic and thermodynamic descriptors, were computed for these substrates in order to obtain a statistical and meaningful QSAR equation. In this sense, by using multiple linear regressions, five mathematical models have been obtained. The best model with only four descriptors (r^2^ = 0.86, Q^2^ = 0.92, S.E.P = 0.38) was validated by the leave-one-out cross-validation method. The antimalarial activity can be explained by the combination of the four mentioned descriptors e.g., electronic potential, dipolar momentum, partition coefficient and molar refractivity. The statistical parameters of this model suggest that it is robust enough to predict the antimalarial activity of new possible compounds; consequently, three small chemical modifications into the structural core of these compounds were performed specifically on the most active compound of the series (compound 13). These three new suggested compounds were leveled as 13A, 13B and 13C, and the predicted biological antimalarial activity is 0.02 µM, 0.03 µM, and 0.07 µM, respectively. In order to complement these results focused on the possible action mechanism of the substrates, a docking simulation was included for these new structures as well as for the compound 13 and the docking scores (binding affinity) obtained for the interaction of these substrates with the cytochrome bc1, were −7.5, −7.2, −6.9 and −7.5 kcal/mol for 13A, 13B, 13C and compound 13, respectively, which suggests that these compounds are good candidates for its biological application in this illness.

## 1. Introduction

Malaria represents a major global public health problem. It is estimated about 200 million people are affected by this illness; most of them belong to tropical and subtropical regions of the world. According to the world Malaria report, nearly 800,000 people died in 2011 because of malaria [1].

Chloroquine and multidrug-resistant *Plasmodium falciparum* strains have spread globally; in addition, there are a number of efficacious drug combinations available. Recently, the artemisinin (the basic component of standard combination for treatment of malaria) resistance has started to emerge; therefore, it is necessary to redouble efforts in order to find novel antimalarial drugs; moreover, cheaper alternatives that can overcome resistance are desperately required [2].

The discovery of antimalarial drugs is a subject of focus in several investigation fields e.g., medicinal chemistry, pharmacokinetics and project planning. Nowadays, computer-assisted searches for drug targets are gaining an extraordinary place because it makes the process of drug discovery more efficient and more cost-effective. In this respect, using in silico approaches such as QSAR studies could play a pivotal role in finding antimalarial drugs [3].

**Figure 1:**
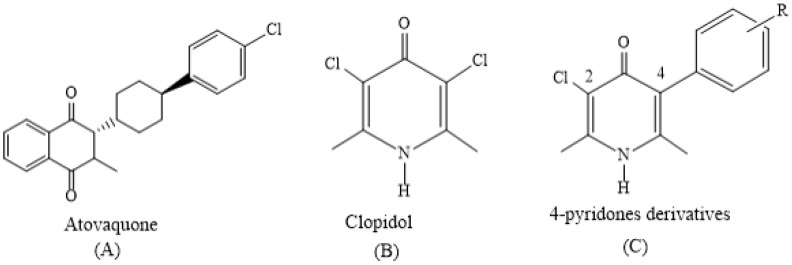
Chemical Structures of antimalarial compounds used in this work. (**A**): Atovaquone; (**B**): Clopidol, C: structural bases of 4-Pyridones derivatives study in this work. Numbering in (**C**) is arbitrary.

The identification of a singular target is fundamental in drug discovery. Once a target is identified, using the molecular docking and QSAR approach, it is easier to gain insight into the mechanisms of drug’s actions and identify potential, new antimalarials. Related to the potential target, the respiratory chain of *P. falciparum* has been pinpointed as an attractive target for chemotherapy. The difference with a mammalian system and the number of drugged pockets inside makes it an extraordinary target to study [4]. In fact, characteristics of the respiratory chain of *P. falciparum* have already borne fruit. Atovaquone, marketed from 1997, (Figure 1A) acts by respiratory chain inhibition and is used in combination with proguanil (malarone, a commercial drugs) to treat multidrug-resistance in areas with chloroquine resistance drugs [4].

The discovery of bc1 protein complex of *P. falciparum* as a target has brought about several attempts to develop new drugs. Currently, those of greatest interest, due to their low toxicity, are new 4,7 Aminocloroquinolines, 4-pyridones, ferrocenes, bisquinolines, and others [5,6,7,8].

The importance of 4-pyridone derivatives as potential antimalarials was increased in 2006 when GlaxoSmithKline (GSK) reported in a preclinical evaluation that a new class of anti-malarial 4(1H) pyridines bind to the bc1 complex of *P. falciparum* [9]. More recently, in an extraordinary and elegant study, Yeates et al. developed a series of di-substituted derivatives of clopidol (Pyridinol anticoccidial agent, Figure 1B). They proved the rational substitution at positions two and four (arbitrary position) can improve the activity in vitro; moreover, the introduction of atovaquone in position four, increased the in vivo efficacy [10].

Previously, the most promising candidate, a non-chiral 4(1H)-pyridone derivative (named GW844520, compound 13 herein) demonstrated high activity against atovaquone-resistant strains and a high selectivity for plasmodium bc1, relative to mammalian bc1. In addition, properties like a short half-life in therapy, a simple chemical synthesis and no resistance was found. Thus, this compound was passed into preclinical development. Unfortunately, drug development of GW844520 was terminated because of the unexpected cardiotoxicity [9].

Even with the unexpected toxicity behavior of GW844520 (compound 13 herein), it was evident that 4-pyridone analogs have significant differences in ADME profile possibility, warranting further investigation of this series; moreover, the change in antimalarial activity due to substitution onto different parts of the compound, suggests the antimalarial activity of 4-pyridone could be highly sensitive to properties of substitutes. Thus, new studies into the influences of electronic effects in 4-pyridone molecules under antimalarial activity are of interest; likewise, the success of new potent antimalarial compounds depends on the knowledge of structural requirements needed to bind the specific target or receptor, or to disrupt the life cycle of plasmodium sp. In this sense, with a potential target already known, QSAR modeling on 4-pyridone represents a good starting point to analyze these characteristics.

Related to antimalarial compounds, several QSAR studies were carried out using different molecular structures. Artemisinin derivatives, cyclic peroxyl ketals, chloroquinoline derivatives, naphthoquinone, naphthoquinone sulfonate, acylate derivatives, sulfonamide derivatives, and sulfonamide derivatives have been studied using QSAR analysis with the goal of finding potential compounds against malaria [11].

Regarding 4-pyridones and related compounds, a few studies have been reported. Nilanjan et al. reported a predictive QSAR approach model by using molecular mechanics (MM). They found that increasing electro topological state atom index (ETSA) as well as frontier electron density of oxygen (carbonyl moiety) and nitrogen atoms might be beneficial for higher antimalarial activity [12].

Basheerulla et al. reported a QSAR study for 4-pyridones using only topological descriptors, taken from semiempirical AM1 geometry. They found that second order valence connectivity index, favoring the activity while having high Burden Eigenvalues decreased the antimalarial activity. According to these two parameters, the author proposed five structures potentially active against *P. falciparum* [13].

The detailed inspection of the works indicated above shows that neither electronic nor thermodynamic parameters were taken into account. Considering the importance of electronic interactions between ligand-receptor (such as hydrogen bonds, London Forces, Van Der Waals forces and so on), and the change in antimalarial activity because of substituting onto different parts of the compound, it seems necessary to carry out new studies that take into account the electronic characteristics of these compounds. In this sense, the quantum chemical calculation (DFT/B3LYP-631++G(dp)) was used in this work to calculate a set of molecular properties of 22 4-pyridone compounds reported by Yeates et al.

## 2. Results and Discussion

**Figure 2:**
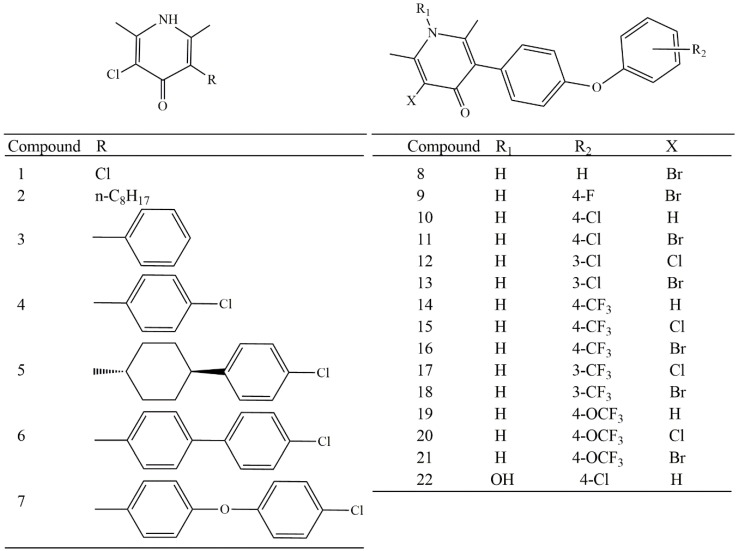
Chemical structures of 4-pyridones studied in this work.

The optimized 3D structures of all compounds shown in Figure 2 were obtained at B3LYP/6-31++G(d,p) level. The second derivative criterion [14] was used to verify that all structures are minimum in the potential energy superficies; in this respect, all compounds studied showed vibrational frequencies >0, proving all of them were minimum energy structures.

Several electronic and thermodynamic molecular descriptors were calculated for all 22 compounds, including the charge in specific positions, named as C2, C4 and N7 (arbitrary numbering).

Using the activity, as pIC_50_ = log(10^6^/IC_50_) as the dependent variable, and all descriptors as independent variables, it was possible to find the most relevant descriptors. According to the procedure, several electronic descriptors (e.g., entropy, s, electronic potential, με, dipole momentum, μ mulliken charge in position2, q2, mulliken charge in N, q7) and three thermodynamic descriptors (Free Energy, G, molar refractivity, MR, and partition coefficient, ClogP) reproduce the antimalarial activity of 4-pyridones studied in this work (Table 1) in a good extension.

Using the descriptors shown in Table 1 and forward selection and backward elimination models, five mathematical models were obtained (filter r > 0.94) (Equations (1)–(4)).

pIC_50_ = 14.97 − 0.0074MR − 0.33μ − 0.602HOMO + 0.5385LUMO − 0.178η − 0.189ω + 3.096q2 r = 0.950; r^2^ = 0.903; σ = 0.23; F = 40.233; σF = 0.0004; Q^2^ = 0.52 (1)

pIC_50_ = 29.94 − 0.875ClogP + 25.939q7 + 0.166μe + 0.126μ + 0.129MR r = 0.960; r^2^ = 0.920; σ = 0.480; F = 37.66; σF = 0.003; Q^2^ = 0.54 (2)

pIC_50_ = 12.383 + 0.263ClogP + 0.0147MR + 0.113μe + 0.162μ r = 0.941 r^2^ = 0.886 σ = 0.38 F = 33.159; σF = 0.0006; Q^2^ = 0.92 (3)

pIC_50_ = 11.98 + 24.930s + 0.017μe +0.388μ + 1.498q2 − 1.070q7 − 0.00012G + 0.317ClogP r = 0.965; r^2^ = 0.930 σ = 0.380; F = 56.686 σF = 5.5 × 10^−7^; Q^2^ = 0.56 (4)

According to statistical results, several equations are acceptable models. However, only Equation (3) has sufficiently important statistical criterion to be considered as a good model, i.e., the ratio molecules/descriptor >5 [15]. Thus, the model described in Equation (3) was chosen as the predictive model of antimalarial activity of 4-pyridones.

**Figure 3:**
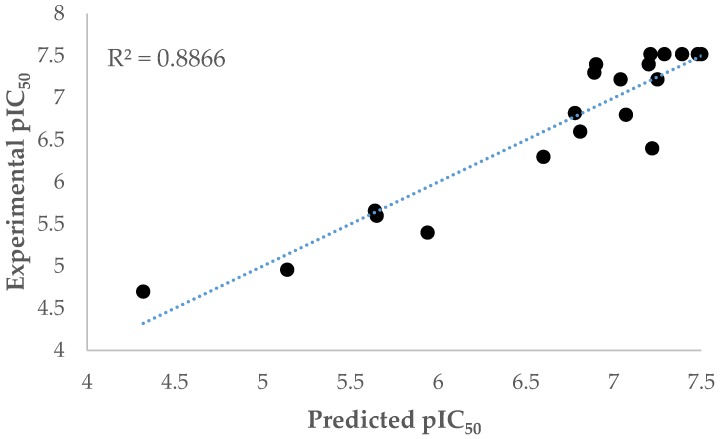
Experimental pIC_50_ vs predicted pIC_50_ using Equation (3).

Table 2 shows the correlation matrix of the molecular descriptor and dependent variable for model 3. Relating to these results, all the independent variables are not correlated with each other. Figure 3 shows an excellent prediction of experimental values using model 3.

All equations were verified and tested through leave-one-out cross-validation method (LOOCV) described before [16]. In agreement with the reported literature [16], a Q^2^ > 0.5 represents a critical value in considering any model as acceptable; therefore, all models are acceptable and model 9, with Q^2^ = 0.923, is statistically validated (Figure 3). 

According to Equation (3), the combination of two electronic descriptors (i.e, µe, and µ) and 2 thermodynamics ones (MR and ClogP), are related with antimalarial activity; moreover, the linear combination of these molecular descriptors could reproduce the in vivo antimalarial activity of 4-pyridones studied herein.

ClogP is a thermodynamic descriptor, defined as the partition coefficient between octanol/agua, hence, a ClogP > 0 indicates the lipophilic character of the substance; otherwise, ClogP < 0 indicates the hydrophilic character of the substance [17,18]. As specified by Equation (3), lipophilic compounds increase the antimalarial activity; in contrast, hydrophilic compounds diminish the antimalarial activity. In this sense, this result supports the proposed idea that the action mechanism of 4-pyridones is the inhibition of cytochrome bc1 in the Qi sites, which is a transmembrane receptor. 4-pyridone compounds with low values of ClogP cannot cross the membrane, and consequently, they are less active. This result provides additional support to the hypothesis of Yeates and coworkers, which suggested that the lipophilic chain in biphenyl-ether moieties increased the biological activity, and they were more active than clopidol.

The electronic chemical potential, µe, is a global index derived from DFT, and is defined as the tendency of any system to gain or lose electrons; a large negative value of µe is related to a good electron acceptor, while a small negative value of µe implies an electron donor [19,20]. Due to the fact that µe is ≤0, and is in agreement with Equation (3), antimalarial activity of 4-pyridones decreases with high values of µe.

Some work reported relates this descriptor with the capability of compounds to form reversible adducts with nucleophiles present in the amino acid chains in proteins. In this respect, Equation (3) suggests an interaction that involves an electronic transfer of 4-pyridones through the pocket of cytochrome bc1 [21,22,23].

Equation (3) shows that molecules with higher dipolar momentum show higher activities. Dipolar momentum is associated with compounds having unsymmetrical electronic density, thus, higher dipolar momentum favors dipole-dipole, cation-π or π-π interactions (Sharma and Rastogi 2007; Lien 1982). In this respect, this result could support the previous experimental investigations, which suggested that antimalarial activity is related to the inhibition of cytochrome bc1, which is highly influenced by the hydrogen bond formation between leucine, histidine and glutamate present in cytochrome bc1 with ligand as atovaquone and clopidol derivatives [24,25,26,27,28,29].

Molar refractivity, MR, is defined as the inverse of molar volume as well as is related to the molecular polarizability [30]. As molar refractivity is associated with molecular volume, and in agreement with Equation (3), electron-withdrawing substituents with higher MR (molar refractivity) or V(W) (van Der Waals volume) are preferred for the activity. The influence of MR over antimalarial activity was already reported [31].

**Figure 4:**
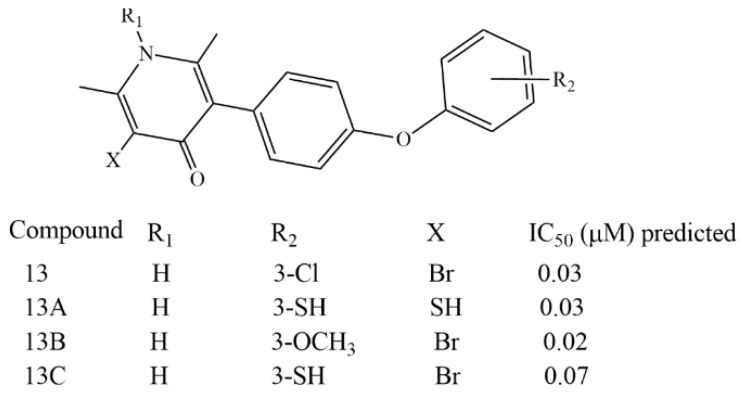
New possible small structure modification on the compound 13 and predicted IC_50_ values.

It is noted that the descriptors found in Equation (3) are understandable and explainable by experimental works already reported. Moreover, the results seem to suggest that, with small structural modifications, new compounds potentially active against malaria could be proposed. Thus, starting from the structure of compound 13 (IC_50_ = 0.03 µM) and using the bioiosteric principle theory [32,33], herein are proposed three new structures with potent antimalarial activity (Figure 4). All compounds were optimized following the same procedure explained above. In the first one, compound 13A, both bromine atoms on the pyridine ring and chlorine atom on the phenoxy ring, were substituted by SH groups, in order to improve the molar refractivity and ClogP. In fact, MR changed from 96 in compound 13 to 104.879 in predicted compound 13A. However, dipole moment and ClogP decreased in the proposed compound; even so, this has similar activity to compound 13. (Predicted IC_50_ = 0.03 µM). In the second proposed structure (13B), only the chlorine atom in the phenoxy moiety was substituted for a methoxy group; this change increased the molar refractivity, dipolar moment and ClogP; consequently, it was predicted that this compound would be more active than compound 13. (Predicted IC_50_ = 0.02 µM). In the last structure (13C), just the chlorine atom was substituted by a SH group. Therefore, there was an increase in both ClogP and molar refractivity; no significant change occurred in the dipole moment or electronic potential. Once again, the group substitution in the specific position results in a potentially active compound but nevertheless is less active than compound 18 (predicted IC_50_ = 0.07 µM) (Figure 4).

**Figure 5:**
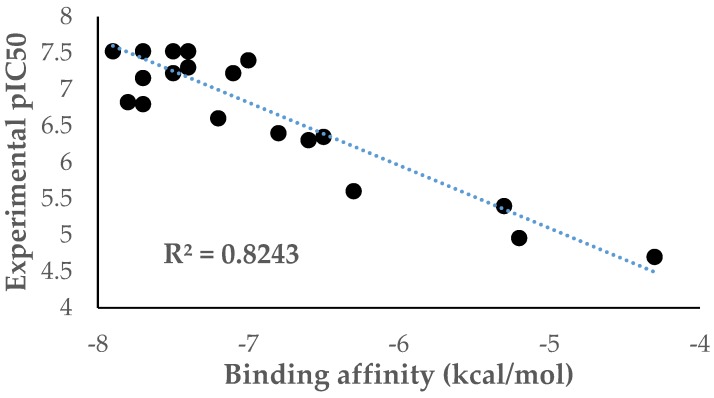
Plot for the binding affinity versus pIC_50._

Based on the results obtained for the structures predicted by Equation (3), it seemed interesting to study the docking properties of these compounds in order to evaluate the action mechanism of these new proposed structures. In this sense, all structures were docked into bc1 complex, specifically at the Qo site (ubiquinol-oxidation pocket), and interestingly a linear correlation was found between the binding affinity and the biological activity (pIC_50_) (R^2^ = 0.824) and also the Atovaquone was considered in the analysis (Figure 5). This result supports the possible action mechanism mainly by the Qo binding site for this series of compounds. Even with the close of this pocket with Fe-S Rieske iron-sulfur, and the presence of heme group, no interaction between ligand and heme group was found.

**Figure 6:**
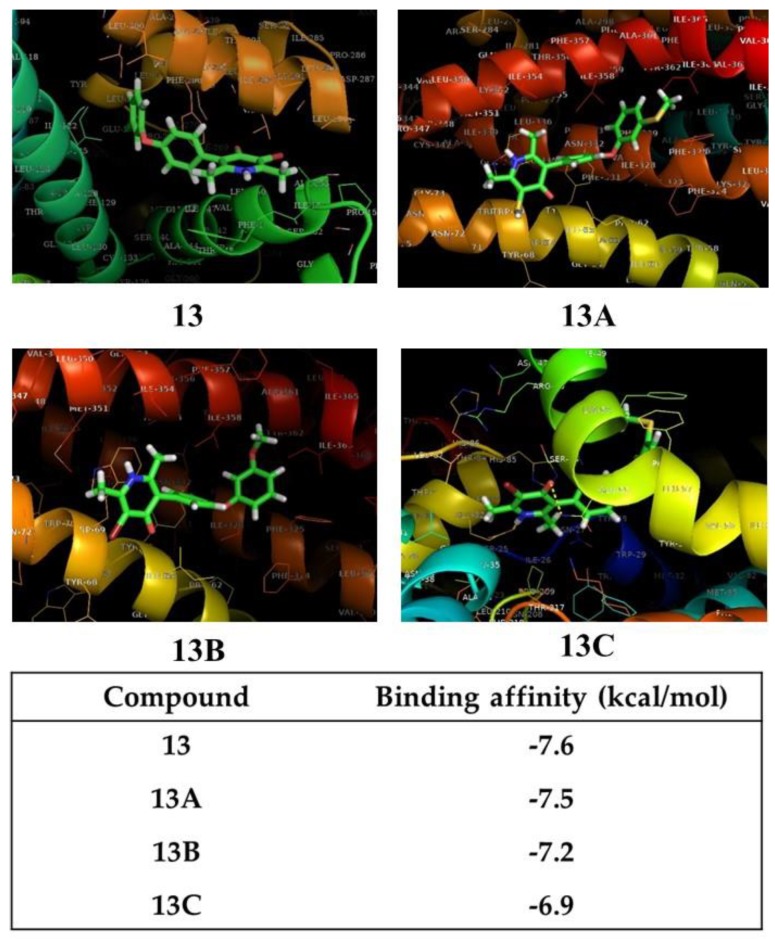
The new antimalarial structure predicted using Equation (3) and docking illustration using the 3D structure of cytochrome bc1 complex.

Based on the results reported above for the docking analysis, it can be used also to validate the three new proposed structure modifications. In this sense, Figure 6 shows the structures of compounds named 13, 13A, 13B and 13C, respectively, and the docking results. The three proposed structures dock very well into the target cytochrome bc1, with suitable score factor coefficients of −7.5, −7.2, and −6.9 kcal/mol for compound 13A, 13B and 13C, respectively. These results suggest that compounds 13A and 13B, which present high negative binding affinity values, can be potential drugs to be synthetized and tested as antimalarial compounds.

In general, the docking calculation suggests a considerable interaction between structure and the amino acid residues leucine, cytosine, glycine, alanine, histidine, glutamate, phenylalanine, and tryptophan. These results suggest the mathematic model (Equation (3)) obtained reproduces the experimental antimalarial activities values of 4-pyridones in an acceptable way; likewise, the chemical descriptors are a suitable guide to rationally modifying the structure of 4-pyridones for reasonable antimalarial drugs design. In addition, Equation (3) reproduces the experimental values and is able to predict new compounds having affinity with *Plasmodium falciparum* cytochrome bc1.

## 3. Materials and Methods

Pharmacological data of in vitro antimalarial activity against T9-94 P. falciparum strain of 4-pyridones were collected from the literature [10]. Twenty-two chemical structures and biological activities (included clopidol) were used. Figure 2 shows the chemical structure, the number and biological activity of all compounds considered in this work.

Molecular optimizations were carried out by using quantum chemical calculations at the DFT/B3LYP level of theory, combined with 6-31++G(d,p) basis set. These structures were characterized as minimum by mean of frequency calculations using Gaussian 09W [34]. The combination of correlation-interchange Becke, Lee, Yang, and Parr (B3LYP) was selected because of its good accuracy level and low computational cost [35,36,37].

The most relevant thermodynamic and electronic properties of 4-pyridones were analyzed by means of conceptual density functional [35]. Molecular descriptors such as vertical Ionization Potential (IP), Electronic Affinity (EA), electronegativity (X), hardness (h), softness (s), electrophilicity index (w) and others, were computed. The IP was calculated by using the energy differences between a radical cation (Ec) and the respective neutral molecule (En) [35]. The EA was calculated as the energy differences between a radical anion (Ea) and the respective neutral molecules (En). The rest of the DFT-based reactivity descriptors were obtained from Equations (5)–(8).

Molecular descriptors such as vertical Ionization Potential (IP), Electronic Affinity (EA), electronegativity (X), hardness (h), softness (s), electrophilicity index (w) Muilliken Charge in specific positions (q), Gibbs energy (G), entropy, (S) entalphy (H), molar refractivity (MR) and ClogP were calculated. The last two descriptors metioned were calculated from Chemaxon Software [38]. The IP was computed by using the energy differences between a radical cation (Ec) and the respective neutral molecule (En) [33]. The EA was calculated as the energy differences between a radical anion (Ea) and the respective neutral molecules (En). The rest of the DFT-based reactivity descriptors were obtained from Equations (5)–(8).

µ = −X = −(IP + EA)/2(5)

η = (IP − EA)/2(6)

S = 1/(2η)(7)

ω = µ^2^/2η (8)

where, (µ): electronic chemical potential; (X): electronegativity.

### 3.1. Statistical Analysis

The more relevant molecular descriptors were selected by taking into account the significance over the dependent variable pIC_50_. In this respect, the descriptors having the greater association with the biological activity (the dependent variable) were used in order to find the mathematical models.

The data of molecular descriptors were studied by means of multiple linear regressions (MLR) [39]. The most relevant properties (e.g., most related to activity) were selected based on the addition-forward selection and backward-elimination methods. The independent variables were individually added or deleted from the model at each step of the regression depending on three criteria: the correlation coefficient (R), the Fisher ratio values (F), and the standard deviation (s). Variables were selected to enter or remove until the best equation was obtained. For this purpose, IBM-SPSS software was used.

Once the QSAR model was found by using theoretical molecular descriptors, it was validated statistically prior to the applications. The best equation was tested for their predictive power using a “leave-one-out cross-validation” procedure [40]. In principle, the so-called “leave one out” approach consists of developing a number of models with one sample omitted at a time. After developing each model, the omitted data is predicted and the differences between actual and predicted “Y” values are calculated. The sum of squares of these differences was computed and, finally, the performance of the model (its predictive ability) can be given by standard error of prediction (*SEP*), defined as:

(9) $$SEP=\sqrt{\frac{\sum_{i=1}^{n}{\left(yi-\mathrm{>ÿ}i\right)}^{2}}{n}}$$

where “*y*” is the experimental value of ln(1/IC_50_), ÿ is the predicted values and *n* is the number of samples used for model building. The predictive ability of the model was also quantified in term of the Q2, defined as:

(10) $$q_{cv}^{2}=1-\frac{\sum_{i=1}^{n}{\left(Y_{exp}-Y_{pred}\right)}^{2}}{\sum_{i=1}^{n}{\left(Y_{exp}-\bar{Y}\right)}^{2}}$$

### 3.2. Docking Analysis

Several researches have pointed out that the cytochrome bc1 complex represents a fundamental target in the lifecycle of *P. falciparum*. Moreover, it is already proved that atovaquone is the only drug, in clinical using, capable of inhibiting the *P. falciparum* bc1 complex [4]. Likewise, Kessl et al. have used the bc1 complex of yeast for studying the interaction of atovaquone with Q0 site of this protein [25]. Even when yeast and *P. falciparum* are two different species, the cytochrome bc1 of *S. Cerevisiae* has been extensively used as a model because of the high sequence identity between its cytochrome bc1 and those corresponding to Plasmodium sp. Moreover, in several works, it has used this protein complex to model the action mechanism of atovaquone and others antimalarial over *P. falciparum* and other pathogens [41,42,43,44,45].

In order to perform an adequate and comparative study, yeast cytochrome bc1 complex was selected as the target in this study; this target was already reported by Lange et al. (Lange et al. 2001) named as 1KB9 on RSC PDB [46]. The target crystal structure was obtained from data bank into mcule 1-clik docking server, which is free available online [46]. The substrates analyzed were saved as .pdb files. Docking simulations were carried out with mcule 1-click Docking server online. The docking scores values obtained for proposed structures are reported and compared with the obtained for already active molecule one (The more negatives values of this scores suggest higher binding energy). The binding site center was established as default, with the Cartesian coordinates (X: 16.6692, Y: −11.3595 and Z: 4.34), and the size of the binding site was 22 Angstrom. This online platform uses Autodock vina as software for the docking simulation.

## 4. Conclusions

All molecular descriptors on 22 antimalarial 4-pyridones were obtained by using the conceptual density functional theory. With these attributes as independent variables and the ln(10^6^/IC_50_) as dependent variables, a modeling process was performed by applying multiple linear regressions. In this sense, four statistically good mathematical models were generated. The best model was validated by the mean of the correlation coefficient, Fischer value, a standard estimation coefficient, and the ration molecules/descriptors >5. The best model, Equation (3), was chosen and validated using leave-one-out-cross-validation method. This model suggests the antimalarial activity could be associated to four molecular descriptors e.g., dipolar moment, electronic potential, partition coefficient and molar refractivity. The dipolar momentum is associated with the interaction via the hydrogen bond between antimalarial compounds and residual amino acids present in the receptor. The electronic potential is related to the possibility to form a reversible complex between 4-pyridones and the receptor cytochrome bc1. The partition coefficient suggests that more lipophilic compounds are most active due to the possible efficiency in the intermembrane crossing. In addition, Equation (3) allowed the proposal of three new potential antimalarial compounds, all of them with high negative docking score values, which suggests high binding affinity with the active center. The model obtained herein, with a low number of manageable descriptors, makes the rational design of new potential drugs against *P. falciparum* easier. However, the combination of theoretical and rational synthesis of these antimalarial compounds is necessary.

## Figures and Tables

**Table 1:** Values of the eight most important properties calculated and their respective values of antimalarial activity.

Comp.	pIC_50_	s	µe	µ	q2	q7	G	MR	ClogP
Clopidol	4.70	3.32	−0.15	8.85	0.04	−0.65	−1321.26	49.35	1.22
2	5.40	3.52	−0.14	7.80	0.04	−0.66	−1175.99	81.12	4.63
3	4.96	3.57	−0.14	7.71	0.05	−0.72	−1092.66	69.01	1.78
4	5.60	3.50	−0.14	8.70	0.05	−0.73	−1552.26	73.82	2.49
5	7.30	3.57	−0.14	9.02	0.04	−0.70	−1786.81	99.52	5.17
6	6.40	3.58	−0.13	8.74	0.07	−0.77	−1783.26	98.95	4.38
7	7.22	3.66	−0.13	8.00	0.06	−0.75	−1858.47	100.06	4.59
8	6.82	3.76	−0.13	7.58	0.02	−0.76	−3510.40	98.07	4.02
9	7.40	3.68	−0.13	8.13	0.01	−0.75	−3609.65	98.29	4.17
10	6.60	3.73	−0.13	8.45	−0.33	−0.76	−1398.87	95.27	3.79
11	7.40	3.67	−0.13	8.41	0.02	−0.76	−3970.00	102.87	4.74
12	7.52	3.63	−0.13	9.70	0.06	−0.75	−1858.47	100.06	4.59
13	7.52	3.63	−0.13	9.60	0.01	−0.76	−3970.00	102.87	4.74
14	6.30	3.43	−0.14	11.19	−0.34	−0.75	−1276.32	96.43	3.96
15	7.22	3.47	−0.14	11.36	0.05	−0.74	−1735.92	101.23	4.76
16	7.52	3.52	−0.13	11.09	−0.01	−0.75	−3847.46	104.04	4.91
17	7.52	3.51	−0.13	10.97	0.06	−0.75	−1735.92	101.23	4.76
18	7.52	3.63	−0.13	9.01	0.01	−0.76	−3886.75	109.08	5.41
19	6.80	3.63	−0.13	10.48	−0.34	−0.75	−1351.54	97.00	4.11
20	7.52	3.57	−0.13	10.52	0.05	−0.74	−1811.14	101.79	4.90
21	7.52	3.59	−0.13	10.36	0.00	−0.75	−3922.67	104.61	5.05
22	5.66	3.65	−0.13	8.67	−0.34	−0.35	−1474.02	0.00	3.69

S: softness; G = Gibbs Energy; µe: electronic chemical potential, (kcal/mol); MR: molar refractivity (cm^3^).

**Table 2:** Correlated matrix for descriptors included in model 3.

Parameter	pIC_50_	ClogP	MR	µ	µ_e_
pIC_50_	1.000	0.850	0.726	0.427	0.647
ClogP	0.850	1.000	0.606	0.352	0.597
MR	0.726	0.606	1.000	0.301	0.217
µ	0.427	0.352	0.301	1.000	−0.035
µ_e_	0.647	0.597	0.217	−0.035	1.000

pIC_50_ = log(10^6^/IC_50_); CLoP: Partition coefficient; MR: molar refractivity, µ: dipolar momentum; µe: Electronic potential.
